# Predictors of fatality including radiographic findings in adults with COVID-19

**DOI:** 10.1186/s12931-020-01411-2

**Published:** 2020-06-11

**Authors:** Kaiyan Li, Dian Chen, Shengchong Chen, Yuchen Feng, Chenli Chang, Zi Wang, Nan Wang, Guohua Zhen

**Affiliations:** 1 Division of Respiratory and Critical Care Medicine, Department of Internal Medicine, Tongji Hospital, Tongji Medical College, Huazhong University of Science and Technology, Wuhan, 430030 China; 2 Key Laboratory of Respiratory Diseases, National Health Commission of People’s Republic of China, Wuhan, China; 3 Department of Radiology, Tongji Hospital, Tongji Medical College, Huazhong University of Science and Technology, Wuhan, 430030 China

**Keywords:** Radiographic findings, Predictor, Fatality, COVID-19

## Abstract

**Background:**

Older age and elevated d-dimer are reported risk factors for coronavirus disease 2019 (COVID-19). However, whether early radiographic change is a predictor of fatality remains unknown.

**Methods:**

We retrospectively reviewed records of all laboratory-confirmed patients admitted to a quarantine unit at Tongji Hospital, a large regional hospital in Wuhan, China, between January 31 and March 5, 2020. Confirmed cases were defined by positive RT-PCR detection of severe acute respiratory syndrome coronavirus 2 (SARS-CoV-2) in throat-swab specimens. Chest CT images were reviewed independently by two radiologists. The Tongji Hospital ethics committee approved this study.

**Results:**

A total of 102 patients were confirmed to have SARS-CoV-2 infection. As of March 25, 85 confirmed patients were discharged, 15 died, and 2 remained hospitalized. When compared with survivors, non-survivors were older (median age, 69 [interquartile range, 58–77] vs. 55 [44–66], *p* = 0.003), and more likely to have decreased lymphocyte count (0.5 vs. 0.9 ×  10^9^/L, *p* = 0.006), elevated lactate dehydrogenase (LDH) (569.0 vs. 272.0 U/L, *p* < 0.001), elevated d-dimer (> 1 μg/mL, 86% vs. 37%, *p* = 0.002) on admission. Older age and elevated LDH were independent risk factors for fatality in a multivariate regression model included the above variables. In a subset of patients with CT images within the first week, higher total severity score, and more involved lung lobes (5 involved lobes) in CT images within the first week were significantly associated with fatality. Moreover, in this subset of patients, higher total severity score was the only independent risk factor in a multivariate analysis incorporating the above mentioned variables.

**Conclusions:**

Older age, elevated LDH on admission, and higher severity score of CT images within the first week are potential predictors of fatality in adults with COVID-19. These predictors may help clinicians identify patients with a poor prognosis at an early stage.

## Background

As of April 2, 2020, there were 823,626 confirmed cases of coronavirus disease 2019 (COVID-19) and 40,598 deaths worldwide [1]. Older age and elevated d-dimer are reported risk factors for COVID-19 [2–4]. However, whether early radiographic change is a predictor of fatality remains unknown.

COVID-19 is caused by a novel coronavirus named severe acute respiratory syndrome coronavirus 2 (SARS­-CoV­-2, previously known as 2019-nCoV). SARS-­CoV-­2 shares similarity with the severe acute respiratory syndrome coronavirus (SARS-CoV) in the disease dynamics, the transmission route, and the cell entry receptors angiotensin-converting enzyme 2 (ACE2) [5–7]. These two viruses are highly homologic to SARS-­like coronaviruses isolated from bats and the genomes of these two virus have 86% identity [5]. Person-to-person transmission of SARS-­CoV-­2 occurs primarily via direct contact or through droplets from an infected individual. SARS­-CoV-­2 targets the respiratory system, leading to pneumonia and respiratory failure in critical patients. Cardiac and renal involvement was also reported [8, 9]. Cytokine storm and systemic inflammatory response syndrome contribute to the pathogenesis of multiple organ failure and coagulation activation in critical patients with COVID-19 [10, 11]. Neutrophil extracellular traps (NETs) is another potential driver of organ damage and mortality in COVID-19 [12]. The most common symptoms at the onset of COVID-19 are fever, cough, fatigue, dyspnea and sputum production [13–16]. The representative chest CT findings on admission were bilateral multiple lobular ground-glass opacity and consolidation [13]. We hypothesized that the extent of the lung lesions in early CT images after symptom onset is a potential predictor of fatality of COVID-19.

In this retrospective cohort study, we reviewed records of 102 laboratory-confirmed patients admitted to a quarantine unit at Tongji Hospital, a large regional hospital in Wuhan, China, between January 31 and March 5, 2020. The demographic, clinical characteristics, laboratory and radiographic findings between survivors and non-survivors were compared and analysed in univariate and multivariate regression models to identify the potential predictors of fatality in COVID-19.

## Methods

### Study population and data collection

This retrospective study was approved by the institutional ethics board of Tongji Hospital of Tongji Medical College of Huazhong University of Science and Technology. Written informed consent was waived. The study included all patients with laboratory-confirmed COVID-19 admitted to a quarantine unit of Tongji Hospital, a large regional hospital in Wuhan, China, between January 31 and March 25, 2020. COVID-19 patients were diagnosed according to World Health Organization (WHO) interim guideline [17]. Confirmed cases were defined by the positive findings in reverse-transcriptase–polymerase-chain-reaction (RT-PCR) assay of throat swab specimens [18]. Clinical characteristics, laboratory test results, and treatment information were extracted from electronic medical records. All laboratory testing and radiological examination were performed according to the clinical care needs of the patient. The criteria for discharge of the patients were relief of clinical symptoms and negative results of two consecutive RT-PCR assays (the time interval for the two assays > 24 h) [19].

### RT-PCR for SARS-CoV-2

Throat swab specimens were tested for SARS-CoV-2 using real-time RT-PCR according to the WHO protocol. The following primers and probes were used for real-time RT-PCR detection of N gene of SARS-CoV-2: N forward primer 5′-GAGCCTTGAATACACCAAAAG-3′, N reverse primer 5′-GCACGATTGCAGCATTGTTAGCAGGATT-3′, N probe 5′-FAMCACATTGGCACCCGCAATCC-MGB-3′. Positive results were confirmed in two independent real-time RT-PCR assays.

### Chest CT protocols and evaluation

High-resolution transverse CT images were obtained using Optima 660 (GE Medical System, Milwaukee, USA) or Somatom Definition AS+ (Siemens Healthineers, Forchheim, Germany). Tube voltage was 100 or 120 kV, and automatic tube current modulation was 100–400 mA. All images were reconstructed with a slice thickness of 1.0 mm or 1.25 mm. The CT images were reviewed by two radiologists (ZW and NW) who were blinded to the final outcome of the patients. Images were reviewed independently. Any disagreements were resolved by discussion and consensus.

A scoring system was used to estimate the extent of lung opacification based on the area involved [20]. Each of the five lung lobes was visually scored from 0 to 5 as: 0, no involvement; 1, < 5% involvement; 2, 5–25% involvement; 3, 26–49% involvement; 4, 50–75% involvement; 5, > 75% involvement. The total severity score was the sum of scores of each lobe, ranging from 0 (no involvement) to 25 (maximum involvement).

### Statistical analysis

Statistical analysis was done with SPSS Statistics Software (version 26; IBM, New York, USA). Continuous variables were presented as median (IQR) and compared using Mann-Whitney U test; categorical variables were presented as number (%) and compared using χ^2^ test or Fisher’s exact test between survivors and non-survivors where appropriate. One-way ANOVA with Bonferroni’s multiple comparison test was performed for comparisons between multiple groups of continuous data. Univariable and multivariable logistic regression models were used to estimate odds ratios and the 95% confidence intervals of the risk factors associated with fatal outcome. A two-sided α of less than 0.05 was considered statistically significant.

## Results

### Demographics and clinical characteristics

A total of 128 patients were admitted. One hundred and two patients were confirmed to have SARS-CoV-2 infection. As of March 25, 85 confirmed patients were discharged, 15 died, and 2 remained hospitalized. The median age was 57 years (interquartile range, 45–70), 59 (58%) were male. The percentage of patients older than 65 years was two-fold higher in non-survivors compared to survivors (Table 1). Forty-four (43%) patients had one or more comorbidities, with hypertension and diabetes mellitus being the most common comorbidity. Coronary heart disease was more frequently observed in non-survivors compared to the survivors (Table 1).

**Table 1:** Demographics and baseline characteristics of patients with COVID-19

	Total (*n* = 102)	Non-survivor (*n* = 15)	Survivor (*n* = 87)	*p* value
**Characteristics**				
Age, years	57 (45–70)	69 (58–77)	55 (44–66)	0.003
< 65	70 (69%)	6 (40%)	64 (74%)	0.010
≥ 65	32 (31%)	9 (60%)	23 (26%)	..
Sex				
Female	43 (42%)	4 (27%)	39 (45%)	0.188
Male	59 (58%)	11 (73%)	48 (55%)	..
Any Comorbidity	44 (43%)	9 (60%)	35 (40%)	0.153
Diabetes	15 (15%)	2 (13%)	13 (15%)	0.871
Hypertension	31 (30%)	7 (47%)	24 (28%)	0.138
Coronary heart disease	4 (4%)	2 (13%)	2 (2%)	0.042
Chronic obstructive pulmonary disease	2 (2%)	1 (7%)	1 (1%)	0.155
Malignancy	5 (5%)	0 (0%)	5 (6%)	1.000
Chronic liver disease	3 (3%)	0 (0%)	3 (3%)	1.000
Other	28 (27%)	5 (33%)	23 (26%)	0.580
Current smoker	7 (7%)	1 (7%)	6 (7%)	0.974
**Symptoms and signs**				
Fever	94 (92%)	14 (93%)	80 (92%)	0.854
Highest temperature, °C	38.6 (38.0–39.0)	38.5 (38.0–38.9)	38.6 (38.0–39.0)	0.458
Chills	23 (23%)	3 (20%)	20 (23%)	0.798
Cough	77 (75%)	13 (87%)	64 (74%)	0.276
Sputum	26 (25%)	6 (40%)	20 (23%)	0.163
Dyspnea	52 (51%)	7 (48%)	45 (52%)	0.717
Hemoptysis	5 (5%)	1 (7%)	4 (5%)	0.732
Chest pain	7 (7%)	1 (7%)	6 (7%)	0.974
Headache	18 (18%)	3 (20%)	15 (17%)	0.796
Fatigue	35 (34%)	5 (33%)	30 (34%)	0.931
Nausea	6 (6%)	1 (7%)	5 (6%)	0.889
Diarrhea	18 (18%)	4 (27%)	14 (16%)	0.321
Myalgia	24 (24%)	3 (20%)	21 (24%)	0.727
Systolic pressure, mm Hg	129.0 (112.0–144.0)	144.0 (126.0–170.0)	127.0 (112.0–141.0)	0.009
Heart rate, beats per minute	93.0 (80.0–103.0)	102.0 (86.0–111.0)	92.0 (80.0–103.0)	0.161
Respiratory rate	20.0 (20.0–24.0)	24.0 (21.0–25.0)	20.0 (20.0–23.0)	0.003
> 20 breaths per min	47 (46%)	12 (80%)	35 (40%)	0.004
Time from symptom onset to hospital admission, days	11.0 (7.0–16.0)	9.0 (6.0–14.0)	11.0 (8.0–18.0)	0.291

Data are median (IQR), n (%), or n/N (%), where N is the total number of patients with available data. *p* values comparing survivor with non-survivor were calculated by χ^2^ test, Fisher’s exact test, or Mann-Whitney U test, as appropriate. COVID-19, coronavirus disease 2019

The most common symptoms were fever (92%), cough (75%), and dyspnea (51%). Tachypnea (respiratory rate > 20 / min) and higher systolic blood pressure were more common in non-survivors compared to survivors (Table 1). The median time between symptom onset and admission was 11 (7–16) days.

### Laboratory findings

When compared with survivors, non-survivors were more likely to have decreased lymphocyte count (0.5 vs. 0.9 × 10^9^/L, *p* = 0.006), thrombocytopenia (60% vs. 8%, *p* < 0.001), elevated lactate dehydrogenase (LDH) (569.0 vs. 272.0 U/L, *p* < 0.001), elevated d-dimer (> 1 μg/mL, 86% vs. 37%, *p* = 0.002), increased hypersensitive troponin I (> 34 pg/mL, 47% vs. 7%, *p* < 0.001), increased NT-proB-type natriuretic peptide (≥ 241 pg/mL, 93% vs. 27%, *p* < 0.001), elevated creatinine (> 104 μmol/L, 40% vs. 8%, *p* = 0.001), elevated blood urea nitrogen (> 9.5 mmol/L, 47% vs. 4%, *p* < 0.001), and increased inflammation markers including C-reactive protein (78.7 vs. 25.4 mg/L, *p* = 0.003) and procalcitonin (≥ 0.05 ng/mL, 100% vs. 54%, *p* < 0.001) (Table 2). Notably, we also observed a significant difference in the expression of inflammation-related cytokines including interleukin (IL)-6, IL-8 and tumor necrosis factor (TNF)-α between the two subsets. The levels of these cytokines were markedly increased in non-survivors compared to survivors (Table 2).

**Table 2:** Laboratory findings of patients with COVID-19 on admission

	Normal range	Total (n = 102)	Non-survivor (n = 15)	Survivor (n = 87)	*p* value
White blood cell count, × 10^9^/L	4.00–10.00	6.0 (4.4–8.6)	9.1 (5.5–11.2)	5.8 (4.4–8.1)	0.011
< 4		11 (11%)	1 (7%)	10 (11%)	0.019
4–10		75 (74%)	8 (53%)	67 (78%)	..
> 10		16 (15%)	6 (40%)	10 (11%)	..
Neutrophil count, × 10^9^/L	1.80–6.30	4.2 (2.9–6.8)	8.0 (3.5–10.6)	4.1 (2.8–6.2)	0.006
Lymphocyte count, × 10^9^/L	1.10–3.20	0.9 (0.6–1.2)	0.5 (0.4–0.8)	0.9 (0.7–1.2)	0.006
< 1·1		66 (65%)	12 (80%)	54 (62%)	0.180
≥1·1		36 (35%)	3 (20%)	33 (38%)	..
Hemoglobin, g/L	130.0–175.0	128.0 (115.0–138.0)	120.0 (110.0–135.0)	128.0 (115.0–139.0)	0.571
Platelet count, × 10^9^/L	125.0–350.0	194.0 (152.0–250.5)	113.0 (97.0–165.0)	206.5 (162.8–267.5)	0.001
< 125		16/101 (16%)	9 (60%)	7/86 (8%)	0.000
≥125		85/101 (84%)	6 (40%)	79/86 (92%)	..
Lactate dehydrogenase, U/L	135–225	294.5 (219.3–417.5)	569.0 (362.0–664.0)	272.0 (205.0–383.0)	0.000
≤225		27 (26%)	1 (7%)	26 (30%)	0.060
> 225		75 (74%)	14 (93%)	61 (70%)	..
D-dimer, μg/mL	≤0·5	0.8 (0.5–1.7)	2.1 (1.2–4.4)	0.7 (0.4–1.5)	0.000
≤0·5		28 (27%)	1 (7%)	27 (31%)	0.002
> 0·5 to ≤1		29 (28%)	1 (7%)	28 (32%)	..
> 1		45 (45%)	13 (86%)	32 (37%)	..
Prothrombin time, s	11.5–14.5	14.2 (13.7–14.8)	14.9 (14.1–17.1)	14.1 (13.6–14.5)	0.001
< 14.5		65 (64%)	4 (27%)	61 (70%)	0.001
≥14.5		37 (36%)	11 (73%)	26 (30%)	..
International Normalized Ratio, INR	0.80–1.20	1.08 (1.04–1.15)	1.16 (1.08–1.37)	1.08 (1.02–1.12)	0.001
Hypersensitive troponin I, pg/mL	≤34.2	5.2 (2.2–16.2)	24.1 (13.0–202.1)	4.3 (2.0–10.6)	0.000
≤34.2		88/101 (87%)	8 (53%)	80/86 (93%)	0.000
> 34.2		13/101 (13%)	7 (47%)	6/86 (7%)	..
NT-proB-type Natriuretic Peptide (BNP), pg/mL	< 241	131.0 (53.5–355.8)	817.5 (348.5–3031.0)	92.5 (42.3–266.5)	0.000
< 241		64/100 (64%)	1/14 (7%)	63/86 (73%)	0.000
≥241		36/100 (36%)	13/14 (93%)	23/86 (27%)	..
Albumin, g/L	35–52	34.8 (31.7–39.5)	31.5 (28.5–34.0)	36.5 (32.5–39.8)	0.002
< 35		52 (51%)	13 (97%)	39 (45%)	0.003
≥35		50 (49%)	2 (13%)	48 (55%)	..
Alanine aminotransferase, U/L	≤41	23.0 (14.0–34.3)	17.0 (13.0–29.0)	23.0 (14.0–35.0)	0.223
≤41		86 (84%)	14 (93%)	72 (83%)	0.298
> 41		16 (16%)	1 (7%)	15 (17%)	..
Aspartate aminotransferase, U/L	≤40	26.0 (19.0–41.8)	34.0 (24.0–54.0)	25.0 (19.0–38.0)	0.187
≤40		75 (74%)	9 (80%)	66 (76%)	0.198
> 40		27 (26%)	6 (20%)	21 (24%)	..
Total bilirubin, μmol/L	≤26	8.5 (6.6–11.6)	8.4 (6.6–15.3)	8.5 (6.6–11.4)	0.752
Creatinine, μmol/L	59–104	68.0 (59.5–84.3)	94.0 (63.0–164.0)	67.0 (58.0–84.0)	0.014
≤104		89 (87%)	9 (60%)	80 (92%)	0.001
> 104		13 (13%)	6 (40%)	7 (8%)	..
Blood urea nitrogen, mmol/L	3.6–9.5	4.6 (3.2–6.2)	9.2 (5.4–13.7)	4.3 (3.2–5.5)	0.000
≤9.5		91 (89%)	8 (53%)	83 (96%)	0.000
> 9.5		11 (11%)	7 (47%)	4 (4%)	..
Potassium, mmol/L	3.50–5.10	4.2 (3.8–4.5)	4.4 (3.6–5.1)	4.1 (3.8–4.5)	0.171
< 3.5		13 (13%)	2 (13%)	11 (13%)	0.012
3.5–5.1		81 (79%)	9 (60%)	72 (83%)	..
> 5.1		8 (8%)	4 (27%)	4 (4%)	..
Sodium, mmol/L	136–145	137.7 (135.8–141.1)	138.6 (133.9–142.6)	137.6 (136.0–141.1)	0.970
< 136		26 (25%)	5 (33%)	21 (24%)	0.248
136–145		74 (73%)	9 (60%)	65 (75%)	..
> 145		2 (2%)	1 (7%)	1 (1%)	..
Calcium, mmol/L	2.20–2.55	2.1 (2.0–2.2)	2.0 (1.9–2.2)	2.1 (2.1–2.3)	0.004
< 2.2		70 (69%)	14 (93%)	56 (64%)	0.026
≥2.2		32 (31%)	1 (7%)	31 (36%)	..
Procalcitonin, ng/mL	< 0.05	0.06 (0.03–0.15)	0.19 (0.12–0.60)	0.05 (0.03–0.10)	0.000
< 0·05		40 (39%)	0 (0%)	40 (46%)	0.000
≥0·05		62 (61%)	15 (100%)	47 (54%)	..
High-sensitivity C-reactive Protein (hs-CRP), mg/L	< 1	34.0 (5.8–86.6)	78.7 (51.3–166.3)	25.4 (3.9–81.3)	0.003
< 3		16 (16%)	0 (0%)	16 (18%)	0.119
≥3		86 (84%)	15 (100%)	71 (82%)	..
IL-1β, pg/ml	< 5	4.9 (4.0–4.9)	4.5 (4.0–4.9)	4.9 (4.0–4.9)	0.388
IL-2R, U/ml	223–710	605.5 (380.8–896.8)	1166.5 (898.8–1788.5)	571.5 (353.0–821.8)	0.001
IL-6, pg/ml	< 7	4.7 (2.2–20.3)	48.4 (12.6–154.1)	4.2 (1.9–16.4)	0.000
IL-8, pg/ml	< 62	10.2 (6.7–19.9)	22.0 (14.0–28.4)	9.3 (6.4–18.6)	0.006
IL-10, pg/ml	< 9.1	4.9 (4.0–4.9)	4.9 (4.0–10.0)	4.9 (4.0–4.9)	0.601
TNF-α, pg/ml	< 8.1	7.5 (5.6–10.1)	13.0 (8.3–23.3)	7.3 (5.6–9.4)	0.006

Data are median (IQR), n (%), or n/N (%), where N is the total number of patients with available data. *p* values comparing survivor with non-survivor were calculated by χ^2^ test, Fisher’s exact test, or Mann-Whitney U test, as appropriated. COVID-19, coronavirus disease 2019

### Radiographic findings

Chest CT is crucial for clinical diagnosis and monitoring temporal changes of the pneumonia caused by SARS-CoV-2. Based on the interval between symptom onset and CT scan, we classified the CT images into 4 stages: week 1 (≤ 7 days after symptom onset), week 2 (> 7 days, ≤ 14 days), week 3 (> 14 days, ≤ 21 days), and week 4 (> 21 days, ≤ 28 days). Twenty-seven survivors had CT images of 3–4 stages. Twenty-one survivors and eleven non-survivors had CT scans within the first week. The demographics, clinical features, and laboratory findings on admission of survivors and non-survivors in this subset were similar to those observed in the entire cohort (Supplementary Tables 1, 2).

**Fig. 1:**
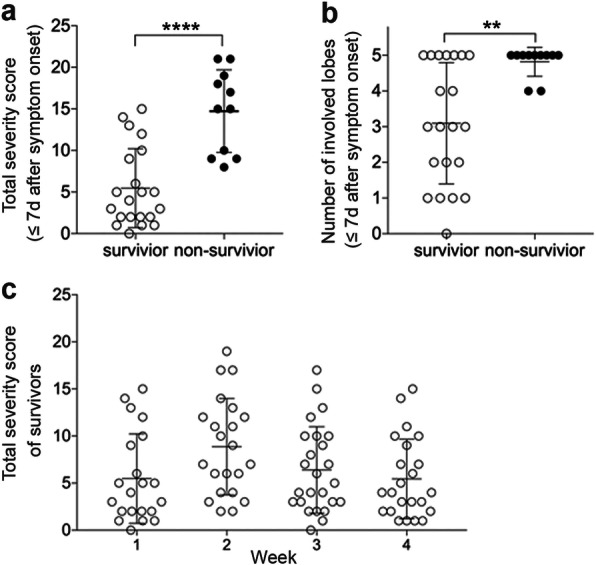
Total severity score and number of involved lung lobes in CT images of survivor and non-survivor. **a-b**, total severity score (**a**) and the number of involved lung lobes (**b**) in CT images of survivors (*n* = 21) and non-survivors (*n* = 11) within the first week (≤7d) after symptom onset. Values of survivors and non-survivors were presented with open and closed circles, respectively. Mann-Whitney U test was used. ****, *p* < 0.0001; **, *p* < 0.01. C. total severity score in CT images of week 1, 2, 3, 4 of the survivors. One-way ANOVA with Bonferroni’s multiple comparison test was used, and there was no significant difference between total severity socres of the 4 weeks

**Fig. 2:**
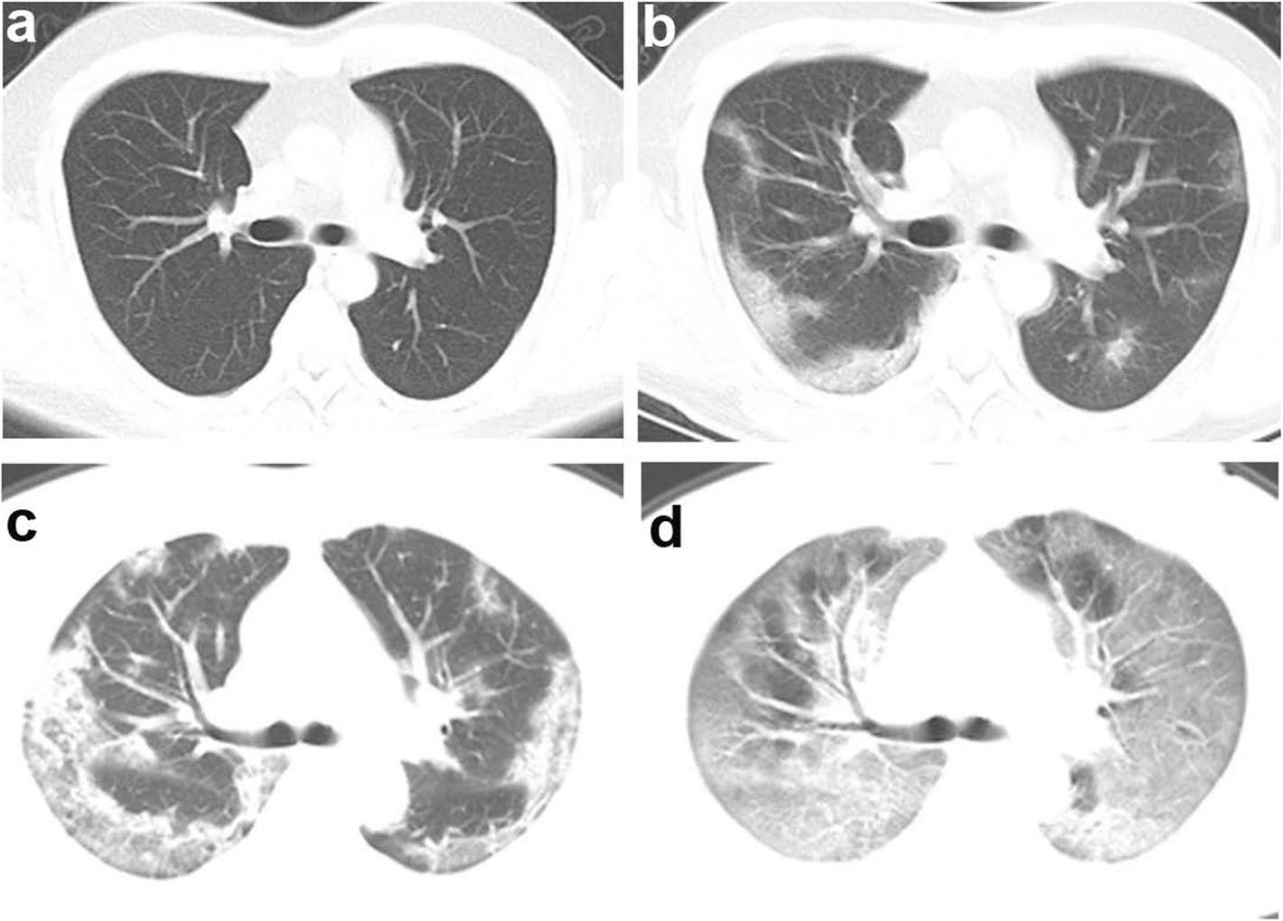
Representative CT images of a survivor and a non-survivor. **a**-**b**, representative transverse CT images of a 38-year-old man with COVID-19 who recovered and was discharged. **a** Normal CT image on the day when the patient had initial symptoms (day 1). **b** day 9 after symptom onset, bilateral and peripheral ground-grass opacity. **c**-**d**, representative transverse CT images of a 41-year-old woman with COVID-19 who died. **c** day 6 after symptom onset, multifocal consolidations and ground-glass opacities affecting the bilateral, subpleural lung parenchyma. **d** day 10 after symptom onset, bilateral extensive ground-glass opacities and consolidation, giving a white lung appearance, with air bronchograms and crazy-paving pattern. The patient died 8 days after this scan

The total severity score and number of involved lung lobes within the first week were significantly greater in non-survivors compared to survivors (Fig. 1a, b, Table 3). Within the first week after symptom onset, two (10%) survivors had normal CT findings, four (19%) survivors had unilateral lung opacification. Fifteen (71%) of survivors whereas eleven (100%) of non-survivors had bilateral lung involvement (Fig. 2). For survivors with serial CT scans performed over 4 weeks, total severity score tended to peak in the second week (Fig. 1c, Table 3).

**Table 3:** CT features of patients with COVID-19

	Week 1 (≤ 7d) after symptom onset		Week 2 (> 7d, ≤ 14d)	Week 3(>14d, ≤ 21d)	Week 4(>21d, ≤ 28d)
	Survivor (n = 21)	Non-Survivor (n = 11)	Survivor(*n* = 22)	Survivor(*n* = 25)	Survivor(*n* = 23)
**Severity score of each lobe**					
Right upper lobe	1 (0–1.5)	3 (2–4)***	2 (1–2)	1 (0.5–2)	1 (0–2)
Right middle lobe	0 (0–1)	2 (1–2)	1 (0–1)	0 (0–1)	0 (0–1)
Right lower lobe	1 (1–3)	3 (3–4)	2.5 (1–3)	1 (1–2.5)	1 (1–2)
Left upper lobe	1 (0–1.5)	3 (1–4)	1.5 (0–3)	1 (0–2)	1 (0–2)
Left lower lobe	1 (0–2.5)	4 (3–5)***	2 (1–3.25)	1 (1–2.5)	1 (0–2)
**Total severity score**	4 (2–9.5)	15 (9–19)****	8 (4–12.25)	5 (3–10)	4 (2–9)
< 15	20 (95.24%)	4 (36.36%)***	19 (86.36%)	23 (92%)	22 (95.65%)
≥ 15	1 (4.76%)	7 (63.64%)	3 (13.64%)	2 (8%)	1 (4.35%)
**Number of involved lobes**	3 (1.5–5)	5 (4–5)**	4.5 (3–5)	4 (3–5)	3 (2–5)
< 5	14 (66.67%)	2 (18.18%)*	11 (50%)	15 (60%)	14 (60.87%)
= 5	7 (33.33%)	9 (81.82%)	11 (50%)	10 (40%)	9 (39.13%)
**Lung involvement**					
No involvement	2 (9.52%)	0 (0%)	0 (0%)	0 (0%)	0 (0%)
Unilateral	4 (19.05%)	0 (0%)	0 (0%)	4 (16%)	3 (13.04%)
Bilateral	15 (71.43%)	11 (100%)	22 (100%)	21 (84%)	20 (86.96%)
**Patterns of opacification**					
Ground glass opacity	20 (95.24%)	10 (90.91%)	21 (95.45%)	18 (72%)	20 (86.96%)
Crazy-paving pattern	2 (9.52%)	5 (45.45%)*	5 (22.72%)	3 (12%)	4 (17.39%)
Consolidation	9 (42.86%)	9 (81.82%)	15 (68.19%)	14 (56%)	1 (4.35%)**
Reticulation	0 (0%)	2 (18.18%)	3 (13.64%)	9 (36%)**	15 (65.22%)****
Pleural effusion	1 (4.76%)	3 (27.27%)	1 (4.55%)	0 (0%)	0 (0%)
**Distribution of opacification**					
No lesion	2 (9.52%)	0 (0%)	0 (0%)	0 (0%)	0 (0%)
Peripheral	10 (47.62%)	8 (72.73%)	13 (59.09%)	18 (72%)	17 (73.91%)
Random	6 (28.57%)	0 (0%)	2 (9.09%)	0 (0%)**	0 (0%)**
Diffuse	3 (14.29%)	3 (21.27%)	7 (31.82%)	7 (28%)	6 (26.09%)
**Time between symptom onset and CT scan**	4 (2.5–5)	5 (2–6)	11.5 (9–13.25)	18 (16–20)	26 (24–28)

Data are median (IQR), or n (%). χ^2^ test, Mann-Whitney U test or Fisher’s exact test were used to compare the data of survivors in week 1 with those of non-survivors, and with the data of survivors in week 2, 3, 4, respectively. One-way ANOVA with Bonferroni’s multiple comparison test was used for comparison of total severity scores of the 4 weeks in survivors. ****, *p* < 0.0001; ***, *p* < 0.001; **, *p* < 0.01; *, *p* < 0.05

The predominant patterns of opacification were bilateral (26 [81%]), peripherally distributed (18 [56%]), ground-glass opacity (30 [94%]), and consolidation (18 [56%]) within the first week (Fig. 2c). The presence of consolidation pattern tended to be more common in non-survivors compared to survivors (82% vs. 43%, *p* = 0.061) at this stage (Table 3). In survivors, the presence of consolidation pattern drastically reduced in the fourth week compared to those in the first week (43% vs. 4%, *p* = 0.003), and the reticulation pattern representing interstitial changes progressively increased in the third and fourth weeks (36%, *p* = 0.002 and 65%, *p* < 0.001, respectively) (Table 3).

### Predictors of fatality

In univariate logistic regression analyses, older age and all of the above mentioned abnormal laboratory findings were significantly associated with fatality. In a multivariate regression model including older age, lymphocytopenia, elevated LDH, and elevated d-dimer, older age (odds ratio per year increase, 1.062 [95% CI 1.005–1.123], *p* = 0.033) and elevated LDH (odds ratio per 1 unit increase, 1.010 [1.005–1.015], *p* < 0.001) were independent risk factors for fatality (Supplementary Table 3).

In the subset of patients with CT images within the first week, using univariate logistic regression analysis, higher total severity score (≥15) (odds ratio, 53 [95% CI 3–369], *p* = 0.003), and more involved lung lobes (5 involved lobes) (odds ratio, 9 [95% CI 2–53], *p* = 0.016) in CT images within the first week were significantly associated with fatality. Moreover, in this subset of patients, higher total severity score (odds ratio per 1unit increase, 1.544 [95% CI 1.004–2.374], *p* = 0.048) was the only independent risk factor in a multivariate analysis incorporating older age, lymphocytopenia, elevated LDH, and elevated d-dimer (Supplementary Table 4).

The treatment for the patients included antibiotics, antivirals, traditional Chinese medicine, corticosteroid, and intravenous immunoglobin (Supplementary Table 5). Non-survivors were more likely to receive corticosteroid compared to survivors (87% vs. 45%, *p* = 0.003). Respiratory supportive therapy includes nasal cannula oxygen therapy, high-flow nasal cannula oxygen therapy, non-invasive and invasive mechanical ventilation (Supplementary Table 5). As expected, non-survivors were more likely to receive non-invasive (87% vs. 10%, *p* < 0.001) and invasive mechanical ventilation (20% vs. 0%, *p* = 0.003). One non-survivor used extracorporeal membrane oxygenation (ECMO), and 2 non-survivors used renal replacement therapy.

## Discussion

Our finding that older age is a risk factor for fatality is consistent with other reports in severe acute respiratory syndrome (SARS), Middle East respiratory syndrome (MERS), and COVID-19 [2–4, 21, 22]. The cell-mediated immune function and humoral immune response were previously reported to be decreased in elderly individuals [23]. Type 2 immune response tend to be predominant in elderly individuals leading to vulnerability to virus infection and poor outcome. Elevated LDH is also a risk factor for poor outcome in SARS [21], which may reflect the diffuse alveolar damage observed in these diseases since high LDH levels were often associated with tissue damage [21, 24]. Elevated d-dimer, a recently reported risk factor for fatality in COVID-19 [2], was associated with fatality with univariate analysis but not with multivariate analysis possibly due to limited sample size of our cohort.

With the CT images within the first week after symptom onset, we were able to analyze the association between early CT changes and fatal outcome. Importantly, we found that the severity score and number of involved lung lobes were associated with fatality in COVID-19. Moreover, higher total severity score was an independent risk factor in a multivariate model. The percentage of lung or number of zones opacified in plain chest radiograph by day 7 were reported to be potential prognostic indicators of fatality in SARS [25]. A recent report showed differences in CT scores between 17 survivors and 10 non-survivors (median time of CT scan after symptom onset 9 and 7.5 days, respectively). However, whether the CT scores at this stage predicted fatality was not analysed in a regression model [26]. Compared to that report, the median time of CT scan after symptom onset (4 and 5 days for survivors and non-survivors, respectively) was earlier in our cohort. Using serial CT images of survivors over 4 weeks after symptom onset, we found that the severity score tended to peak at the second week. In survivors, the presence of consolidation pattern significantly reduced in the fourth week, while the reticulation pattern representing interstitial changes progressively increased in the third and fourth week.

Lymphocytopenia and elevated hypersensitive troponin I were also associated with fatal outcome in our cohort. In community acquired pneumonia, lymphocytopenia was reported to be associated with fatality [27]. Lymphocytopenia in patients with COVID-19 can be explained by impaired lymphogenesis or increased apoptosis, increased adhesion to vascular endothelium, or massive migration of lymphocytes to the lungs [28]. Of note, we found that coronary heart disease was more common as a comorbidity in non-survivors compared to survivors. Consistently, elevated hypersensitive troponin I on admission was more common in non-survivors compared to survivors. Older age, cardiovascular disease, and the severity of pneumonia were reported as risk factors of cardiac events for patients with acute pneumonia [29].

Cytokine storm may play a vital role in the pathogenesis of critical COVID-19 patients. Elevated levels of inflammatory biomarkers and infiltrated immune cells in lung lesion were recently reported in critical COVID-19 patients [30]. Previous reports on SARS-COV showed elevated proinflammatory cytokines in serum including IL-8, MCP-1, IP-10, IFN-γ, IL-12, IL-1β and IL-6, leading to the recruitment of alveolar macrophages and extensive lung damage [31]. An increased plasma level of IFN-α2, IFN-γ, TNF-α, IL-10, IL-15 and IL-17 was reported during the acute phase of MERS-CoV infection, presenting a prominent Th1 and Th17 cytokine profile [32]. Similarly, in our cohort, non-survivors had markedly higher levels of high-sensitivity C-reactive protein, procalcitonin, IL-2R, IL-6, IL-8 and TNF-α. Thus, corticosteroids could be an effective treatment for critical COVID-19 patients to alleviate inflammation-related acute lung injury.

Our study had several limitations. Firstly, this is a retrospective cohort study and the accuracy of the data were dependent upon the medical records. Due to the limited sample size, observation bias may exist in the study. Secondly, there could be a selection bias in the multivariate analysis for the risk factors.

## Conclusion

This report suggests that older age, elevated LDH on admission, and extensive lung lesions in early CT images are potential predictors of fatal outcome in adults with COVID-19. These predictors may help clinicians identify patients with a poor prognosis at an early stage.

## Supplementary information

**Additional file 1 **Supplementary Appendix. **Table S1**. Demographics and baseline characteristics of the subset of patients included in the analysis of CTs performed within the first week after symptom onset. **Table S2**. Laboratory findings of the subset of patients included in the analysis of CTs performed within the first week after symptom onset. **Table S3**. Risk factors associated with fatality. **Table S4**. Risk factors associated with fatality of the subset of patients included in the analysis of CTs performed within the first week after symptom onset. **Table S5**. Treatments and outcomes. **Table S6**. Treatments and outcomes of the subset of patients included in the analysis of CTs performed within the first week after symptom onset.

## Data Availability

All data generated or analysed during this study are included in this published article and its supplementary information files.

## Abbreviations

COVID-19: Coronavirus disease 2019
SARS-CoV-2: Severe acute respiratory syndrome coronavirus 2
SARS: Severe acute respiratory syndrome
ACE2: angiotensin-converting enzyme 2
MERS: Middle East respiratory syndrome
ECMO: Extracorporeal membrane oxygenation
ICU: Intensive care unit
IQR: Interquartile ranges
OR: odds ratio
SPSS: Statistical product and service solutions
