# Supplementary material for: Predictors of fatality including radiographic findings in adults with COVID-19
Source: Respir Res. 2020 Jun 11;21:146. doi: 10.1186/s12931-020-01411-2 (PMC7289230; doi:10.1186/s12931-020-01411-2)
Supplement: Supplementary file 1 — Additional file 1 Supplementary Appendix. Table S1. Demographics and baseline characteristics of the subset of patients included in the analysis of CTs performed within the first week after symptom onset. Table S2. Laboratory findings of the subset of patients included in the analysis of CTs performed within the first week after symptom onset. Table S3. Risk factors associated with fatality. Table S4. Risk factors associated with fatality of the subset of patients included in the analysis of CTs performed within the first week after symptom onset. Table S5. Treatments and outcomes. Table S6. Treatments and outcomes of the subset of patients included in the analysis of CTs performed within the first week after symptom onset. [file 12931_2020_1411_MOESM1_ESM.pdf]

Supplementary Appendix

Predictors of Fatality including Radiographic Findings in Adults with COVID-19

Kaiyan Li, Dian Chen, Shengchong Chen, Yuchen Feng, Chenli Chang, Zi Wang, Nan Wang, Guohua Zhen

Table of contents

Table S1. Demographics and baseline characteristics of the subset of patients included in the analysis of CTs performed within the first week after symptom onset…………2

Table S2. Laboratory findings of the subset of patients included in the analysis of CTs performed within the first week after symptom onset……………………………....4

Table S3. Risk factors associated with fatality…………………………………………7

Table S4. Risk factors associated with fatality of the subset of patients included in the analysis of CTs performed within the first week after symptom onset…………………9

Table S5. Treatments and outcomes…………………………………………………..10

Table S6. Treatments and outcomes of the subset of patients included in the analysis of CTs performed within the first week after symptom onset……………………………11

Table S1. Demographics and baseline characteristics of the subset of patients included in the analysis of CT within the first week after symptom onset

	Total (n=32)	Non-survivor (n=11)	Survivor (n=21)	p value
**Characteristics**				
Age, years	58(40-70)	69(57-78)	51(33-70)	0.020
<65	20 (62%)	5 (45%)	15 (71%)	0.149
≥65	12 (38%)	6 (55%)	6 (29%)	
Sex				0.266
Female	13 (41%)	3 (27%)	10 (48%)	
Male	19 (59%)	8 (73%)	11 (52%)	
Any Comorbidity	13 (41%)	6 (55%)	7 (33%)	0.246
Diabetes	5 (16%)	2 (18%)	3 (14%)	0.773
Hypertension	9 (28%)	5 (45%)	4 (19%)	0.115
Coronary heart disease	1 (3%)	1 (9%)	0 (0%)	0.344
Chronic obstructive pulmonary disease	1 (3%)	1 (9%)	0 (0%)	0.344
Malignancy	0 (0%)	0 (0%)	0 (0%)	..
Chronic liver disease	1 (3%)	0 (0%)	1 (5%)	1.000
Other	8 (25%)	4 (36%)	4 (19%)	0.283
Current smoker	3 (9%)	1 (9%)	2 (10%)	0.968
**Symptoms and signs**				
Fever	30 (94%)	10 (91%)	20 (95%)	0.631
Highest temperature, °C	38.6(38.0-39.0)	38.6(37.6-39.0)	38.5(38.0-39.0)	0.611
Chills	7 (22%)	3 (27%)	4 (19%)	0.593
Cough	23 (72%)	9 (82%)	14 (67%)	0.365
Sputum	8 (25%)	5 (45%)	3 (14%)	0.053
Dyspnea	16 (50%)	5 (45%)	11 (52%)	0.710
Hemoptysis	2 (6%)	1 (9%)	1 (5%)	0.631
Chest pain	2 (6%)	1 (9%)	1 (5%)	0.631
Headache	3 (9%)	1 (9%)	2 (10%)	0.968
Fatigue	8 (25%)	2 (18%)	6 (29%)	0.519
Nausea	1 (3%)	0 (0%)	1 (5%)	1.000
Diarrhea	5 (16%)	3 (27%)	2 (10%)	0.189
Myalgia	7 (22%)	3 (27%)	4 (19%)	0.593
Systolic pressure, mm Hg	129.0(114.0-144.0)	144.0(125.0-171.0)	126.0(112.0-139.0)	0.074
Heart rate, beats per minute	95.0(81.0-110.0)	103.0(86.0-111.0)	86.0(80.0-109.0)	0.367
Respiratory rate	20.0(20.0-24.0)	24.0(20.0-25.0)	20.0(20.0-22.0)	0.022
>20 breaths per min	14 (44%)	8 (73%)	6 (29%)	0.017
Time from symptom onset to hospital admission, days	10.0(7.0-13.0)	8.0(6.0-14.0)	10.0(7.0-13.0)	0.639

Data are median (IQR), n (%), or n/N (%), where N is the total number of patients with available data. χ^2 test, Fisher's exact test, or Mann-Whitney U test were used to compare the values between survivors and non-survivors as appropriate.

Table S2. Laboratory findings of the subset of patients included in the analysis of CT within the first week after symptom onset

	Normal range	Total (n=32)	Non-survivor (n=11)	Survivor (n=21)	p value
White blood cell count, × 10^9/L	4.00-10.00	5.2(4.2-8.3)	9.1(6.2-11.8)	4.7(4.1-5.4)	0.000
<4		4 (13%)	0 (0%)	4 (19%)	0.002
4–10		23 (72%)	6 (55%)	17 (91%)	
>10		5 (15%)	5 (45%)	0 (0%)	
Neutrophil count, × 10^9/L	1.80-6.30	3.5(2.8-6.9)	8.0(3.5-11.3)	2.9(2.7-4.3)	0.000
Lymphocyte count, × 10^9/L	1.10-3.20	0.8(0.5-1.2)	0.7(0.4-1.5)	0.9(0.6-1.2)	0.307
<1·1		22 (69%)	8 (73%)	14 (67%)	0.725
≥1·1		10 (31%)	3 (27%)	7 (33%)	
Hemoglobin, g/L	130.0-175.0	126.5(120.0-140.0)	120.0(110.0-135.0)	129.0(122.5-141.5)	0.113
Platelet count, × 10^9/L	125.0-350.0	162.0(124.0-242.0)	124.0(87.0-242.0)	175.0(133.8-287.0)	0.066
<125		8/31 (26%)	6 (55%)	2/20 (10%)	0.007
≥125		23/31 (74%)	5 (45%)	18/20 (90%)	
Lactate dehydrogenase, U/L	135-225	328.0(197.5-541.8)	570.0(440.0-671.0)	261.0(194.5-379.5)	0.001
≤225		9 (28%)	1 (9%)	8 (38%)	0.083
>225		23 (72%)	10 (91%)	13 (62%)	
D-dimer, μg/mL	≤0·5	0.9(0.5-2.0)	2.3(1.6-22.0)	0.6(0.4-0.9)	0.000
≤0·5		8 (25%)	1 (9%)	7 (33%)	0.000
>0·5 to ≤1		11 (34%)	0 (0%)	11 (52%)	
>1		13 (41%)	10 (91%)	3 (15%)	
Prothrombin time, s	11.5-14.5	14.8(13.8-15.4)	15.5(14.8-17.7)	14.3(13.4-15.0)	0.005
<14.5		14 (44%)	2 (18%)	12 (57%)	0.035

≥14.5		18 (56%)	9 (82%)	9 (43%)	
International Normalized Ratio, INR	0.80-1.20	1.15(1.05-1.20)	1.21(1.14-1.45)	1.09(1.01-1.17)	0.005
Hypersensitive troponin I, pg/mL	≤34.2	11.0(4.2-27.0)	23.1(13.0-204.7)	6.0(2.3-13.2)	0.005
≤34.2		24/31 (77%)	6 (55%)	18/20 (90%)	0.024
>34.2		7/31 (23%)	5 (45%)	2/20 (10%)	
NT-proB-type Natriuretic Peptide (BNP), pg/mL	<241	192.0(71.0-702.5)	777.0(348.5-2847.0)	90.5(58.8-194.5)	0.000
<241		17/30 (57%)	0 (0%)	17/20 (85%)	0.000
≥241		13/30 (43%)	10 (100%)	3/20 (15%)	
Albumin, g/L	35-52	33.4(30.1-39.5)	31.5(28.2-34.2)	36.0(32.2-39.9)	0.034
<35		18 (56%)	9 (82%)	9 (43%)	0.035
≥35		14 (44%)	2 (18%)	12 (57%)	
Alanine aminotransferase, U/L	≤41	19.5(13.0-28.5)	17.0(14.0-29.0)	20.0(12.0-27.5)	0.725
≤41		28 (87%)	10 (91%)	18 (86%)	0.673
>41		4 (13%)	1 (9%)	3 (14%)	
Aspartate aminotransferase, U/L	≤40	30.0(19.3-43.0)	34.0(24.0-54.0)	24.0(19.0-36.0)	0.271
≤40		24 (75%)	7 (64%)	17 (81%)	0.283
>40		8 (25%)	4 (36%)	4 (19%)	
Total bilirubin, μmol/L	≤26	7.9(6.2-10.3)	7.5(6.6-9.8)	8.1(6.0-10.9)	1.000
Creatinine, μmol/L	59-104	68.0(61.3-87.8)	100.0(62.0-164.0)	67.0(59.5-76.0)	0.088
≤104		27 (84%)	6 (55%)	21 (100%)	0.002
>104		5 (16%)	5 (45%)	0 (0%)	
Blood urea nitrogen, mmol/L	3.6-9.5	4.7(3.3-8.4)	9.2(5.4-13.7)	4.0(3.3-5.2)	0.008
≤9.5		26 (81%)	6 (55%)	20 (95%)	0.005
>9.5		6 (19%)	5 (45%)	1 (5%)	
Potassium, mmol/L	3.50-5.10	4.1(3.5-4.5)	4.4(3.6-5.3)	4.0(3.5-4.3)	0.208
<3.5		7 (22%)	2 (18%)	5 (24%)	0.042

3.5-5.1		22 (69%)	6 (55%)	16 (76%)	
>5.1		3 (9%)	3 (27%)	0 (0%)	
Sodium, mmol/L	136-145	139.0(136.4-141.9)	138.7(137.2-142.6)	139.1(135.6-141.7)	0.938
<136		7 (22%)	2 (18%)	5 (24%)	0.363
136-145		24 (75%)	8 (73%)	16 (76%)	
>145		1 (3%)	1 (9%)	0 (0%)	
Calcium, mmol/L	2.20-2.55	2.1(2.0-2.2)	2.0(1.9-2.2)	2.1(2.0-2.2)	0.081
<2.2		25 (78%)	10 (91%)	15 (71%)	0.205
≥2.2		7 (22%)	1 (9%)	6 (29%)	
Procalcitonin, ng/mL	<0.05	0.05(0.03-0.18)	0.32(0.11-0.60)	0.04(0.02-0.06)	0.000
<0·05		17 (53%)	1 (9%)	16 (76%)	0.000
≥0·05		15 (47%)	10 (91%)	5 (24%)	
High-sensitivity C-reactive Protein (hs-CRP), mg/L	<1	52.2(9.6-114.7)	102.1(51.3-194.8)	27.0(5.5-71.1)	0.009
<3		4 (13%)	0 (0%)	4 (19%)	0.272
≥3		28 (87%)	11 (100%)	17 (81%)	
IL-1β, pg/ml	<5	4.9(4.7-4.9)	4.9(4.2-4.9)	4.9(4.7-4.9)	0.652
IL-2R, U/ml	223-710	514.0(292.5-744.5)	1076.5(671.8-1699.5)	454.5(270.3-563.0)	0.005
IL-6, pg/ml	<7	4.5(1.4-22.6)	65.1(11.3-154.1)	3.3(1.4-16.7)	0.019
IL-8, pg/ml	<62	10.2(6.1-18.9)	27.6(14.1-64.9)	9.4(5.7-15.9)	0.010
IL-10, pg/ml	<9.1	4.9(4.0-4.9)	8.3(4.9-17.0)	4.9(4.0-4.9)	0.081
TNF-α, pg/ml	<8.1	7.0(4.9-9.6)	21.3(13.2-28.9)	5.7(3.8-7.9)	0.000

Data are median (IQR), n (%), or n/N (%), where N is the total number of patients with available data. χ^2 test, Fisher's exact test, or Mann-Whitney U test were used to compare the values between survivors and non-survivors as appropriate.

Table S3. Risk factors associated with fatality

	Univariable OR (95% CI)	p value	Multivariable OR (95% CI)	p value
**Demographics and clinical characteristics**				
Age, years*	1.07(1.02-1.11)	0.006	1.062(1.005-1.123)	0.033
<65	1 (ref)	..	..	..
≥65	4.17(1.34-13.02)	0.014	..	..
Female sex (vs male)	0.45(0.13-1.52)	0.196	..	..
Comorbidity present (vs not present)				
Diabetes	0.88(0.18-4.34)	0.871	..	..
Hypertension	2.30(0.75-7.03)	0.120	..	..
Coronary heart disease	6.54(0.85-50.54)	0.072	..	..
Other	1.39(0.43-4.50)	0.582	..	..
Current smoker	0.96(0.11-8.63)	0.974		
**Laboratory findings**				
White blood cell count, × 10^9/L	1.29(1.07-1.56)	0.008	..	..
<4	0.84(0.09-7.43)	0.873	..	..
4–10	1 (ref)	..	..	..
>10	5.03(1.44-17.54)	0.011	..	..
Neutrophil count, × 10^9/L*	1.33(1.11-1.59)	0.002	..	..
Lymphocyte count, × 10^9/L*	0.17(0.04-0.79)	0.024	..	..
Platelet count, × 10^9/L	0.98(0.97-0.99)	0.001	..	..
<125	16.93(4.66-61.51)	0.000	..	..
≥125	1 (ref)	..	..	..
Lactate dehydrogenase, U/L*	1.01(1.00-1.02)	0.000	1.010(1.005-1.015)	0.000
≤225	1 (ref)	..	..	..
>225	5.97(0.75-47.77)	0.092	..	..
D-dimer, μg/mL	1.14(1.04-1.25)	0.007	..	..
≤0·5	1 (ref)	..	..	..
>0·5 to ≤1	0.96(0.06-16.21)	0.980	..	..
>1	10.97(1.35-89.34)	0.025	..	..
Prothrombin time, s	1.08(0.96-1.21)	0.210	..	..
<14.5	1 (ref)	..	..	..
≥14.5	6.45(1.88-22.14)	0.003	..	..
Hypersensitive troponin I, pg/mL	1.00(0.99-1.01)	0.313	..	..
≤34.2	1 (ref)	..	..	..
>34.2	11.67(3.15-43.26)	0.000	..	..
NT-proB-type natriuretic peptide (BNP), pg/mL	1.00(1.00-1.00)	0.706	..	..

<241	1 (ref)	..	..	..
≥241	35.61(4.41-287.68)	0.001	..	..
Albumin, g/L	0.82(0.71-0.93)	0.003	..	..
<35	8.00(1.70-37.60)	0.008	..	..
≥35	1 (ref)	..	..	..
Alanine aminotransferase, U/L	0.97(0.93-1.02)	0.204	..	..
≤41	1 (ref)	..	..	..
>41	0.34(0.04-2.81)	0.319	..	..
Creatinine, μmol/L	1.00(0.99-1.01)	0.535	..	..
≤104	1 (ref)	..	..	..
>104	7.62(2.10-27.68)	0.002	..	..
Blood urea nitrogen, mmol/L	1.12(1.02-1.23)	0.018	..	..
≤9.5	1 (ref)	..	..	..
>9.5	18.16(4.36-75.62)	0.000	..	..
Procalcitonin, ng/mL*	1.86(0.82-4.20)	0.138	..	..
High-sensitivity C-reactive Protein (hs-CRP), mg/L*	1.01(1.00-1.02)	0.005	..	..
IL-2R, U/ml*	1.003(1.001-1.004)	0.001	..	..
IL-6, pg/ml*	1.02(1.01-1.04)	0.002	..	..
TNF-α, pg/ml*	1.19(1.06-1.34)	0.005	..	..

OR=odds ratio. *Per 1 unit increase.

Table S4. Risk factors associated with fatality of the subset of patients with CT severity scores within the first week after symptom onset

	Univariable OR (95% CI)	p value	Multivariable OR (95% CI)	p value
**Demographics**				
Age, years*	1.06(1.01-1.13)	0.027	..	..
**Laboratory findings on admission**				
Lymphocyte count, × 10^9/L*	0.53(0.09-3.03)	0.474	..	..
Lactate dehydrogenase, U/L	1.01(1.00-1.02)	0.003	..	..
≤225	1 (ref)	..	..	..
>225	6.15(0.66-57.60)	0.111	..	..
D-dimer, µg/mL	9.30(1.85-46.74)	0.007	..	..
≤0·5	1 (ref)	..	..	..
>0·5 to ≤1	..	..	..	..
>1	23.33(1.99-273.29)	0.012	..	..
Hypersensitive troponin I, pg/mL	1.00(0.99-1.01)	0.411	..	..
≤34.2	1 (ref)	..	..	..
>34.2	7.50(1.14-49.26)	0.036	..	..
**CT findings within the first week after symptom onset**				
Total severity score*	1.39(1.12-1.72)	0.003	1.544(1.004-2.374)	0.048
<15	1 (ref)	..	..	..
≥15	35.00(3.32-368.57)	0.003	..	..
Number of involved lung lobes	1.366(1.003-1.860)	0.048	..	..
<5	1 (ref)	..	..	..
=5	9.00(1.52-53.40)	0.016	..	..

OR=odds ratio. *Per 1 unit increase.

Table S5. Treatments and outcomes

	Total (n=102)	Non-survivor (n=15)	Survivor (n=87)	p value
**Treatments**				
Antibiotics	81 (79%)	15 (100%)	66 (76%)	0.036
Antiviral treatment	75 (74%)	7 (47%)	68 (78%)	0.011
Traditional Chinese medicine	79 (77%)	3 (20%)	76 (87%)	0.000
Corticosteroids	52 (51%)	13 (87%)	39 (45%)	0.003
Intravenous immunoglobin	19 (19%)	5 (33%)	14 (16%)	0.113
Nasal cannula oxygen therapy	76 (75%)	5 (33%)	71 (82%)	0.000
High-flow nasal cannula oxygen therapy	6 (6%)	2 (13%)	4 (5%)	0.184
Non-invasive mechanical ventilation	22 (22%)	13 (87%)	9 (10%)	0.000
Invasive mechanical ventilation	3 (3%)	3 (20%)	0 (0%)	0.003
ECMO	1 (1%)	1 (7%)	0 (0%)	..
Renal replacement therapy	2 (2%)	2 (13%)	0 (0%)	0.020
**Outcomes**				
Respiratory failure	35 (34%)	15 (100%)	20 (23%)	0.000
Heart failure	7 (7%)	4 (27%)	3 (3%)	0.001
Acute kidney failure	5 (5%)	5 (33%)	0 (0%)	0.000
ICU admission	3 (3%)	3 (20%)	0 (0%)	0.003
Hospital length of stay, days	21(13-29)	11(7-13)	22(16-30)	0.000
Time from symptom onset to ICU admission, days	19(21-34)	19(21-34)	..	..
ICU length of stay, days	9(3-20)	9(3-20)	..	..
Time from symptom onset to death or discharge, days	34(25-42)	22(17-26)	35(28-44)	0.000

Data are median (IQR) or n (%). p values were calculated by χ^2 test, or Fisher's exact test, as appropriate. ECMO=extracorporeal membrane oxygenation. ICU=intensive care unit.

Table S6. Treatments and outcomes of the subset of patients included in the analysis of CT within the first week after symptom onset

	Total (n=32)	Non-survivor (n=11)	Survivor (n=21)	p value
**Treatments**				
Antibiotics	29 (91%)	11 (100%)	18 (86%)	0.188
Antiviral treatment	25 (78%)	5 (45%)	20 (95%)	0.001
Traditional Chinese medicine	21 (66%)	1 (9%)	20 (95%)	0.000
Corticosteroids	19 (59%)	9 (82%)	10 (48%)	0.061
Intravenous immunoglobin	7 (22%)	3 (27%)	4 (19%)	0.593
Nasal cannula oxygen therapy	19 (59%)	2 (18%)	17 (81%)	0.001
High-flow nasal cannula oxygen therapy	0 (0%)	0 (0%)	0 (0%)	..
Non-invasive mechanical ventilation	13 (41%)	10 (91%)	3 (14%)	0.000
Invasive mechanical ventilation	0 (0%)	0 (0%)	0 (0%)	..
ECMO	0 (0%)	0 (0%)	0 (0%)	..
Renal replacement therapy	0 (0%)	0 (0%)	0 (0%)	..
**Outcomes**				
Respiratory failure	18 (56%)	11 (100%)	7 (33%)	0.000
Heart failure	3 (9%)	2 (18%)	1 (5%)	0.216
Acute kidney failure	4 (13%)	4 (36%)	0 (0%)	0.009
ICU admission	0 (0%)	0 (0%)	0 (0%)	..
Hospital length of stay, days	17(11-26)	10(7-12)	23(16-30)	0.000
Time from symptom onset to ICU admission, days	0 (0%)	0 (0%)	0 (0%)	..
ICU length of stay, days	0 (0%)	0 (0%)	0 (0%)	..
Time from symptom onset to death or discharge, days	28(22-35)	20(16-25)	34(27-38)	0.000

The counting data were presented as count (percentage of the total). p values were calculated by χ^2 test, or Fisher's exact test, as appropriate.
